# The Abdominal Adiposity Index (A Body Shape Index) Predicts 10-Year All-Cause Mortality in Elderly Active Non-Obese Subjects

**DOI:** 10.3390/jcm13206155

**Published:** 2024-10-16

**Authors:** Alessio Nunnari, Filippo Giorgio Di Girolamo, Kaja Teraž, Nicola Fiotti, Boštjan Šimunič, Filippo Mearelli, Rado Pišot, Gianni Biolo

**Affiliations:** 1 Unit of Internal Medicine, Clinica Medica, Department of Medical Surgical and Health Sciences, University of Trieste, Strada di Fiume 447, 34100 Trieste, Italy; kaja.teraz@zrs-kp.si (K.T.); fiotti@units.it (N.F.); filippome@libero.it (F.M.); gianni.biolo@asugi.sanita.fvg.it (G.B.); 2 Department of Medicine, Surgery and Health Sciences MD, University of Trieste, Strada di Fiume 447, 34100 Trieste, Italy; 3 SC Assistenza Farmaceutica, Cattinara Hospital, Azienda Sanitaria Universitaria Giuliano Isontina, Strada di Fiume 447, 34100 Trieste, Italy; 4 Institute for Kinesiology Research, Science and Research Centre Koper, Garibaldijeva ulica 1, 6000 Koper, Slovenia; bostjan.simunic@zrs-kp.si (B.Š.); rado.pisot@zrs-kp.si (R.P.)

**Keywords:** ABSI, a body shape index, body shape, free fat mass, sarcopenia, prognostic index, elderly, mortality

## Abstract

**Background/Objectives**: A Body Shape Index (ABSI), which accounts for waist circumference relative to mass and height, shows a robust association with mortality risk. The present study evaluates the effectiveness of ABSI as a predictor of 10-year all-cause mortality in physically active, non-obese elderly individuals. **Methods**: This prospective cohort study included 159 volunteers (94 women, aged 60–80 years), recruited in the frame of the “Physical Activity and Nutrition for Great Ageing” (PANGeA) Cross-border Cooperation Program Slovenia–Italy 2007–2013, and followed for 10 years. Baseline characteristics included anthropometric measurements, bioelectrical impedance analysis, and cardiovascular fitness tests (VO_2_max). Statistical analyses (Cox regression, Kaplan–Meier survival) were conducted to examine the relationship between ABSI and mortality. **Results**: During the 10-year follow-up, 10 deaths (6.7%) were recorded. ABSI (adjusted for age, smoking, comorbidities, and therapy) was an independent predictor of mortality (hazard ratio = 4.65, *p* < 0.001). Higher ABSI scores were linked to reduced VO_2_max (r = −0.190, *p* = 0.017) and increased systolic blood pressure (r = 0.262, *p* = 0.001). An ABSI-based predictive model showed strong discriminatory power (AUROC = 0.91). **Conclusions**: ABSI is a reliable predictor of 10-year mortality in active, non-obese elderly individuals and may improve risk stratification in clinical practice.

## 1. Introduction

Hypertension, tobacco use, high blood glucose, obesity, and physical inactivity are recognized as major modifiable risk factors for cardiovascular, cerebrovascular, and oncological pathologies [1]. In recent years, central adiposity has shown a better correlation with mortality than assessments based solely on individual weight. It has emerged as a primary predictor of many metabolic abnormalities and increased mortality [2]. Visceral fat tissue, interspersed with resident immune cells, when activated, increases local or systemic inflammation, leading to the production of cytokines and other immune and pro-inflammatory mediators, promoting insulin resistance, oxidative stress, and altered cell metabolism. Abdominal fat accumulation is associated with changes in glucose and lipid metabolism [3], primarily due to insulin resistance, resulting in hyperlipidemia, hypertension [4], glucose intolerance, and mitochondrial abnormalities in skeletal muscle [5].

With age, fat deposition shifts away from subcutaneous fat, while intramuscular and visceral fat depots modify muscle architecture and performance [6,7]. This shift contributes to a progressive and generalized loss of skeletal muscle mass [8]. The decline in muscle mass and metabolic capacity facilitates the conversion of calories into fat, creating a vicious cycle of fat gain and reduced mobility due to excessive weight and muscle loss [9,10], ultimately resulting in sarcopenic obesity.

As reported in many studies, age-related changes in muscle composition and obesity are synergistically associated with general functional decline. In fact, sarcopenic obesity is linked to higher mortality [6,7,8,9,10,11,12,13], largely because of changes in body composition favoring fat mass over muscle mass, along with a decline in cardiorespiratory fitness [12]. Changes in muscle composition due to fat accumulation play an important role in reducing cardiorespiratory fitness and, consequently, increasing mortality risk [13,14,15,16,17]. Furthermore, excess visceral fat accumulation, through multifactorial pathogenesis [18], is associated with hypertension [4,5,6,7,8,9,10,11,12,13,14,15,16,17,18,19].

An individual’s leanness or corpulence is commonly assessed using the Body Mass Index (BMI), but this measure does not account for fat distribution or differentiate between fat and muscle mass [19,20]. Therefore, clinicians have explored alternative anthropometric measurements that better reflect body composition and mortality risk [10,11,12,13,14,15,16,17,18,19,20,21], such as waist circumference (WC) and waist-to-hip and waist-to-height ratios [22]. Most of these measures fail to reflect body composition effectively or are easily affected by variations in other body measurements [10]. A new body shape index (ABSI) has been introduced as an anthropometric measure unrelated to BMI, based on waist circumference adjusted for weight and height.

ABSI offers a better explanation of how central abdominal adiposity is strongly associated with mortality than other anthropometric measurements, and it captures additional harmful effects not captured by BMI [3,4,5,6,7,8,9,10,11,12,13,14,15,16,17,18,19,20,21,22,23,24].

The aim of this study is to better understand the effects of abdominal adiposity, measured by ABSI, on health and cardiorespiratory decline in a population of non-obese elderly individuals and to evaluate whether ABSI could be used as a predictor of higher mortality risk in active, non-obese elderly subjects.

## 2. Materials and Methods

The present study is part of a larger research project, Physical Activity and Nutrition for Healthy Ageing (PANGeA) [24], conducted in accordance with the latest revision of the Declaration of Helsinki. Both the baseline and follow-up measurements were approved by the University Ethics Committee of the University of Trieste (Report No. 49 from the meeting held on 10 June 2013). Additionally, the clinical trial protocol was registered on ClinicalTrials.gov (Identifier: NCT04899531).

### 2.1. Participants

On 25 and 26 June 2014, 159 elderly participants (94 women and 65 men), who were non-obese, physically active, and without significant health problems, were selected in Trieste as part of the Cross-border Cooperation Program Slovenia–Italy, 2007–2013, PANGeA: Physical Activity and Nutrition for Healthy Ageing (ClinicalTrials.gov ID NCT04899531) [24]. Participants were considered physically active if they were capable of performing daily activities independently. Their physical activity level was assessed using a questionnaire, and informed consent was obtained from each participant.

A Case Report Form (CRF) was completed, detailing their health status, comorbidities (e.g., active smoking, solid or hematologic malignancy in remission, metabolic disorders like dyslipidemia or metabolic syndrome), and home therapy. At the time of enrollment, all comorbidities were either in remission or well-controlled. To simplify data collection, the participants could select their home therapy from predefined categories, though the transcription of specific medications was discretionary. Beta-blockers were the most commonly reported class of antiarrhythmic drugs. All data were stored in a database, with sensitive information replaced by a randomized identity code.

**Figure 1:**
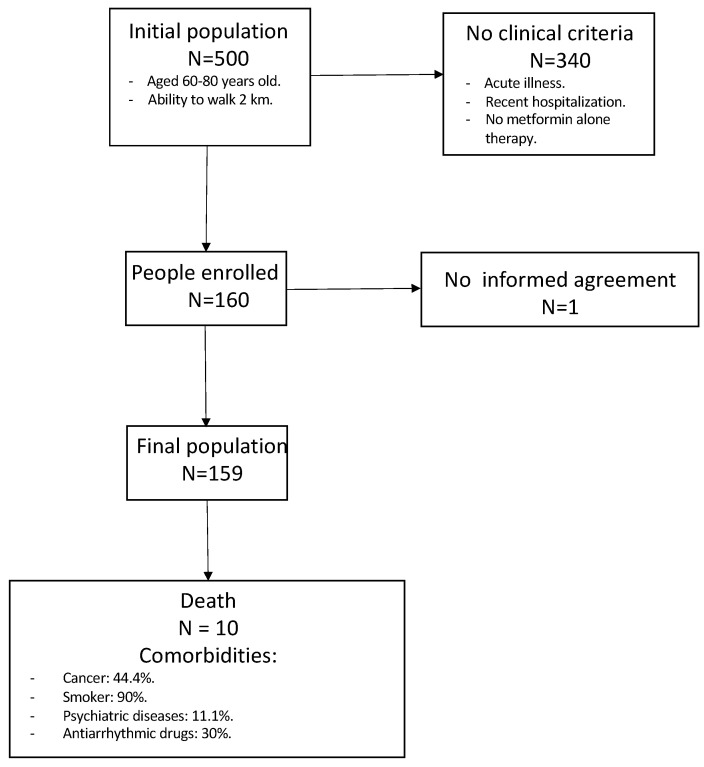
Flow diagram of the inclusion and exclusion criteria.

The inclusion criteria required participants to be aged 60–80 and capable of walking 2 km independently. The exclusion criteria included severe cognitive decline (MoCA score < 10 after adjustment for age and education), recent hospitalization (<6 months), acute illness, or diabetes requiring insulin therapy (except for metformin). In 2024, a 10-year survival follow-up was conducted using a regional registry system (Figure 1). Because of Italian privacy laws, electronic health records were reviewed only for participants who provided written consent for access to their electronic records, meaning no health data updates were available for the majority.

### 2.2. Baseline Assessment

We initially conducted a cycle ergometer test on 33 subjects, while those unable to ride completed a 2 km walking test, to estimate VO_2_max (maximum oxygen consumption) as follows:

VO_2_max = Q × Δ(a − v).

The arterial alveolar oxygen difference was measured by capillary sampling from the earlobe. The following anthropometric indices were also measured: weight, height, and BMI (Body Mass Index), which was calculated using the formula mass/height^2^. Waist circumference (WC) was measured at the uppermost border of the iliac crests. ABSI was calculated using the following equation [3]:

ABSI = WC/(BMI^2/3^ × H^½^).

where WC and H (height) were expressed in meters. Body composition was evaluated using BIA (bioimpedance analysis) with a Tanita model BC418MA. BIA measurements were performed in the morning with an empty bladder, after an overnight fast. Bioimpedance analysis was conducted with subjects in a supine position. The following values were obtained: Reactance (Xc) and Resistance (Rc). The estimation of FFM (fat-free mass) and FM (fat mass) was calculated based on these two values using the software provided by the manufacturer, which includes specific equations for different BMI ranges. FFMI (Fat-Free Mass Index) was calculated using the following equation:

FFMI = FFM/H^2^.

where FFM represents fat-free mass and H represents height in meters. Arterial blood pressure and glycemia were considered, and muscle strength was measured using hand grip strength. This was assessed by gripping a dynamometer with the dominant hand while seated, with the arm at a 90° angle. The average of three measurements was recorded.

### 2.3. Statistical Analysis

All statistical analyses were performed using SPSS 28.0 and R Studio 2022.02.0 + 443. All continuous variables were checked for distribution, and errors in data entry were imputed with the median. For variables that were not normally distributed, a logarithmic transformation was performed. Variables with scales that were difficult to process were standardized using Z-values, calculated as follows:

Z=(X−μx)/σx.

where X represents an individual value, μ represents the mean, and σ represents the standard deviation. In descriptive tables, all continuous variables were expressed as medians and interquartile ranges, while all qualitative variables were expressed as absolute values and percentages. In these tables, missing values were deleted using listwise selection, while in other tables, missing data were processed by pairwise selection. The covariance and variance for continuous variables were analyzed using Analysis of Variance (ANOVA) and General Linear Models Univariate analysis (GLM Univariate). The dependence between categorical variables was evaluated using Pearson’s test or Fisher’s exact test for variables with fewer than 10 cases. The Mann–Whitney U non-parametric test was used for continuous variables that were not normally distributed. Correlation was analyzed using Pearson’s test for normally distributed variables and Spearman’s non-parametric test for other continuous variables. We built a logistic model by including all variables with a statistical significance of *p* < 0.1. The model’s power to discriminate mortality in our population was assessed using Receiver Operating Characteristic (ROC) analysis and compared, using the Z statistic, with the ABSI index standardized on the average. The statistical significance of Kaplan–Meier curves was calculated with the Log-Rank test. Risk factors and hazard ratios were identified using Cox regression.

## 3. Results

Our study population consisted primarily of females (59.7%) with a median age of 67 years, a BMI of 25 (22–25), an ABSI of 0.081 (0.079–0.084), and an estimated fat-free mass of 66 kg (60–70) who were active smokers (50%), with hypertension (29.6%), and on antihypertensive (28.3%) and hypolipidemic agents (14.5%) (Table 1).

In the ANOVA and univariate generalized linear model analyses, female gender (*p* = 0.002), age (*p* < 0.001), maximum systolic pressure (*p* < 0.001), VO_2_Max (*p* = 0.017), thyroid medication (*p* = 0.048), and 10-year mortality (*p* = 0.003) were found to be independent variables for ABSI (Table 2).

As mentioned earlier, we standardized ABSI using the z-score, which was found to be correlated with VO_2_Max (r = −0.190; *p* = 0.017) in Spearman’s correlation test and with maximum systolic pressure (r = 0.262; *p* = 0.001) in Pearson’s correlation (Table 3).

BMI (*p* = 0.033), waist circumference (*p* = 0.007), standardized ABSI (*p* = 0.005), smoking dependence (*p* = 0.018), cancer (*p* = 0.012), psychiatric diseases (*p* = 0.067), and antiarrhythmic drugs (*p* = 0.017) were identified as independent variables differentiating survivors from non-survivors in terms of 10-year mortality (Table 4).

Using logistic regression, standardized ABSI, antiarrhythmic drugs, cancer, and smoking showed odds ratios for 10-year mortality of 2.771, 20.706, 20.272, and 23.260, respectively (Table 5).

**Figure 2:**
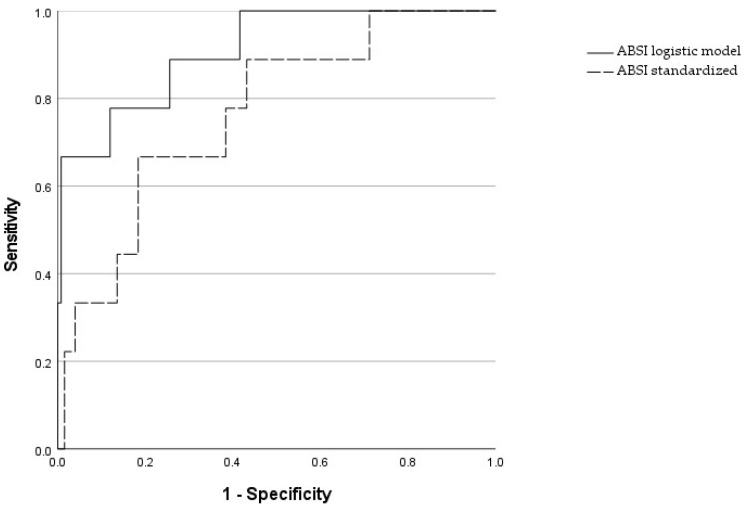
Comparison of the standardized ABSI alone and the logistic regression model with ABSI, cancer, cigarette use, and pharmacological therapy for heart rhythm: Receiver Operating Characteristic for predicting 10-year mortality.

The logistic model had an AUROC of 0.91 (with a PPV of 85.71% and an NNV of 97.64%), while the ABSI standardized model alone had an AUROC of 0.76 (see Figure 2 and Table 6).

The Z score between curves was significantly different (*p* = 0.003) (Table 7).

**Figure 3:**
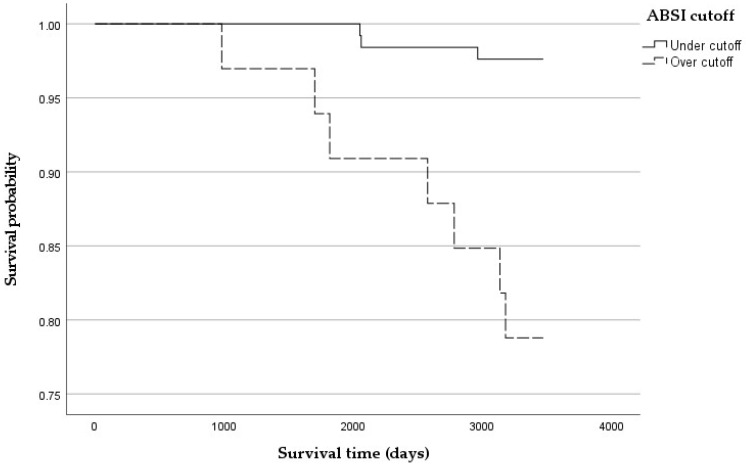
Kaplan–Meier curves for mortality risk at 10 years stratified for the ABSI cutoff of 0.77.

An ABSI standardized maximized cutoff for 10-year mortality of 0.77 was identified, with a PPV of 21% and an NPV of 97%. This value was used to draw stratified Kaplan–Meier curves (Figure 3), with a Log Rank test result of less than 0.001 (Table 8).

A proportional hazard assumption test for each independent variable was performed, and then we executed a Cox regression proportional hazards model where the ABSI cutoff had a hazard ratio of 4.650, the use of antiarrhythmic drugs had a hazard ratio of 13.862, cancer had a hazard ratio of 9.456, and smoke dependence had a hazard ratio of 12.047 (Table 9).

In the post hoc analysis, we divided the population by sex and opted to use the lean mass index, which, after excluding gender-related variability, is more sensitive to differences in physical structure.

Age (*p* = 0.001), systolic blood pressure (*p* = 0.001), VO_2_Max (*p* = 0.001), and hand grip (*p* = 0.028) were independent of the male gender. Furthermore, we performed the logarithmic transformation for FFMI values (Table 10).

**Figure 4:**
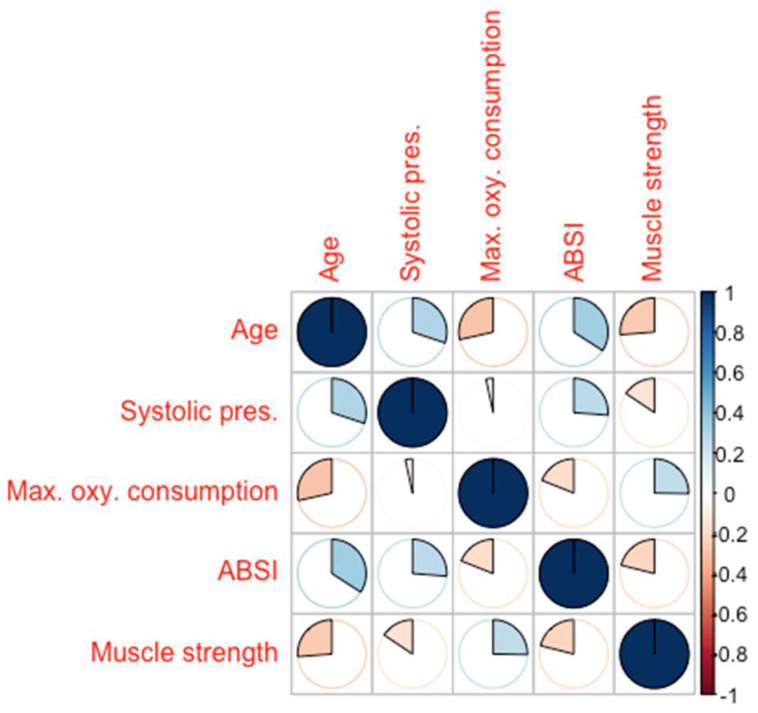
Correlation matrix of ABSI with age, systolic pressure, maximum oxygen consumption, and muscle strength. Warm colors indicate a negative correlation, while cold colors indicate a positive correlation; the strengths of the correlations can be deduced from the pie charts.

In our male population, VO_2_Max (r = −0.460, *p* = 0.001), hand grip (r = −0.273, *p* = 0.028), systolic blood pressure (r = 0.274, *p* = 0.001), and age (r = 0.431, *p* = 0.001) were correlated with ABSI, as shown in the correlation matrix (Figure 4).

For the female group, age (*p* = 0.005) was independently associated with ABSI (Table 11). Furthermore, it had a direct correlation with standardized ABSI (r = 0.251; *p* = 0.015).

## 4. Discussion

BMI, together with waist and hip circumference, used to be considered the foundation for assessing individual mortality. However, since 2012, ABSI has been validated for studying body mass constitution [21]. In recent publications, ABSI has shown superior performance in studies on frail patients [25], as well as in research on mortality in cancer [26,27] and cardiovascular disease patients [28], with minimal dependence on gender and age differences.

In our research, we hypothesized that ABSI could be an independent risk factor for long-term mortality in a population without significant active comorbidities. We performed a 10-year follow-up for 159 physically active, non-obese elderly subjects, without active comorbidities, enrolled in a previous study [3].

Our population consists of a majority of females (59.7%), with a mean age of 67 years, active smokers (50%), and slightly overweight individuals, with medians of 0.081 for ABSI and 25 for BMI, and an estimated free fat mass of 65 kg. The principal comorbidity is hypertension (29.6%), with 28.3% undergoing treatment and 14.5% associated with hypolipidemic therapy. Ten of them died (6.7%) at the 10-year follow-up.

For all statistical analyses other than demographic assessments, we used the standardized ABSI [20]. Of all variables independently associated with ABSI (female gender, age, free fat mass, maximum systolic pressure, VO_2_max, hand grip strength, headache, urinary disorder, anxiety, number of medications, therapy for hypertension, thyroid issues, and arrhythmias), only maximum arterial pressure (r = 0.262; *p* < 0.001) and VO_2_Max (r = −0.190; *p* = 0.017) were correlated. This result could be attributed to the effects of beta-blockers and other antihypertensives on muscular metabolism.

The survival analysis found that there are no substantial differences between survivors and non-survivors in demographic variables. In the non-survivor group, we found higher BMI (28 vs. 25), waist circumference (101 vs. 89 cm), glycemia (5.77 vs. 5.27 mmol/L), and standardized ABSI (0.90 vs. −0.06). The most frequent comorbidities were cigarette smoking (90% vs. 48%), cancer (44.4% vs. 9.6%), the need for drugs to control arrhythmias (30% vs. 4.7%), and psychiatric diseases (11.1%). Of all these variables, standardized ABSI, former cigarette smoking, cancer, and the use of drugs for heart rhythm control were independent factors in multivariate analysis.

We evaluated the ability of these risk factors to determine mortality using a logistic regression model. The AUROC was found to be 0.91 with a PPV of 85.71% and an NPV of 97.64%.

Using an ABSI cutoff of 0.77, we divided the population into two groups to study survival using Kaplan–Meier curves. The group above the cutoff showed higher mortality (Log Rank *p* < 0.001). Then, combining the identified cutoff with significant clinical variables, we performed a Cox regression and obtained the following hazard ratios (HRs): ABSI cutoff HR: 4.650; need for antiarrhythmic drugs HR: 13.862; cancer HR: 9.456; and smoke dependence HR: 12.047.

The risk–benefit ratio of antiarrhythmic therapy was markedly higher in cardiac patients and, therefore, not adequately evaluated in our population. Thus, the only two modifiable risk factors liable for prevention were ABSI and smoking.

In the end, we conducted a post hoc analysis by gender. Unfortunately, the sample size was insufficient to determine differences in mortality. However, in the male population, standardized ABSI showed a correlation with VO_2_max (r = −0.460; *p* = 0.001), hand grip strength (r = −0.273; *p* = 0.028), systolic blood pressure (r = 0.274; *p* = 0.001), and age (r = 0.431; *p* = 0.001), further supporting the evidence that ABSI, unlike BMI, is an index closely related to muscle mass in men.

In the female population, the only independent variable was age (*p* = 0.005), with a correlation coefficient of 0.251 (*p* = 0.015).

Our research has limitations such as the lack of randomization, sample size issues, and possible selection bias in the population studied. It should be considered that unlike studies conducted by other research groups, selecting a non-obese population without comorbidities impacting quality of life is a strength of our study. Additionally, the effect of comorbidities was further reduced by allowing the use of a screened population.

The lack of randomization was mitigated by enlisting volunteers through mass media outreach rather than pre-selection processes.

It should be emphasized that future evaluations using a validation cohort could strengthen our results and diminish the limitations mentioned above.

## 5. Conclusions

Our research confirms the importance of ABSI as an anthropometric variable in estimating mortality risk. Although this finding has already been pointed out in various studies involving specific pathologies, we demonstrated its relevance in an active population without specific comorbidities.

ABSI is found to be correlated with VO_2_Max, consequently showing its ability to indirectly estimate aerobic capacity.

It is noteworthy that age is the only correlated variable in both the male and female populations. However, only in the male population does standardized ABSI correlate with the following muscle mass-dependent variables: VO_2_Max, muscle strength, and maximum systolic pressure.

These correlations should reflect the relationship between standardized ABSI and fitness levels and visceral adiposity. It may also serve as an important estimator of sarcopenic obesity due to a catabolic-pro-inflammatory environment leading to protein catabolism and muscle wasting, which influences systolic pressure values [29].

The finding that age is the unique clinical variable associated with standardized ABSI in females could be due to menopause effects [30], while in men, this variable involves changes in physical structure related to testosterone levels.

The ABSI cutoff and smoking addiction are the only modifiable risk factors identified in our population that warrant active prevention efforts. Based on the current literature, testosterone therapy [31] is currently the only treatment shown to increase lean body mass. Although controlled protein nutrition has been found useful for reducing sarcopenia, it has shown no utility in modifying ABSI.

In conclusion, since ABSI has been shown to be more accurate than BMI in stratifying death risk, it should be used as a new parameter in outpatient populations. Additionally, its inclusion in integrated medical records should be evaluated [7].

## Figures and Tables

**Table 1:** All variables are expressed with their associated units of measurement (^a^). Categorical variables are presented as absolute values with percentages indicated in parentheses (^b^). Continuous variables are presented with the median and interquartile range indicated in parentheses (^c^).

**Characteristics**	
N°	159
Female	94 (59.7) ^b^
Age (years) ^a^	67 (64–70) ^c^
Survivor	149 (93.7)
**Clinical variables**	
BMI (kg/m^2^)	25 (22–25)
Waist circumference (cm)	89 (83–96)
ABSI (m^1/2^/kg^2/3^)	0.081 (0.079–0.084)
Hand grip (kg)	29.8 (25.0–43.5)
VO_2_MAX (mL/kg/min)	23.38 (20.79–25.24)
FFM (kg)	66 (60–70)
**Comorbidities**	
Former cigarette smoking	80 (50.3)
Asthma	8 (5)
Allergy	32 (20.1)
COPD	10 (6.3)
COPD exacerbation	8 (5)
IMA	1 (0.6)
Hypertension	47 (29.6)
Ischemic stroke	0
Rheumatoid arthritis	14 (8.8)
Arthrosis	32 (20.1)
Low back pain	54 (34)
Neck pain	34 (21.4)
Cephalgia	12 (7.5)
DM	4 (2.5)
Gastro duodenal ulcers	8 (5)
Liver cirrhosis	4 (2.5)
Cancer	16 (10.1)
Urinary disorders	26 (17)
Anxiety	12 (7.5)
Depression	3 (1.9)
Psychiatric disorders	1 (0.6)
Impairment post-major trauma	5 (3.1)
Metabolic disorders	30 (18.9)
**Therapy**	
Number of drugs taken	1 (0–2)
Diuretics	11 (6.9)
TP Dyslipidemia	23 (14.5)
TP Diabetes	2 (1.3)
TP Hypertension	45 (28.3)
TP Gout	1 (0.6)
NSAIDs	9 (5.7)
TP Gastro duodenal ulcer	9 (5.7)
TP Anti-arrhythmias	10 (6.3)
TP Thyroid	11 (6.9)
TP Asthma/allergy	4 (2.5)
CCS	0
Antidepressant	2 (1.3)
Other therapy	44 (27.7)

BMI = Body Mass Index; ABSI: a new body shape index; VO_2_Max = maximum oxygen consumption; FFM = free fat mass; COPD = chronic obstructive pulmonary disease; IMA = myocardial infarction; CCS = systemic corticosteroids; DM = diabetes mellitus; NSAIDs = nonsteroidal anti-inflammatory drugs.

**Table 2:** *p*-Values indicate statistical significance obtained through the generalized linear model (GLM) for quantitative variables and analysis of variance (ANOVA) for qualitative variables (^a^) (^b^).

ABSI	*p*-Value ^a,b^
**Demographic variables**	
Female	0.002
Age (years)	<0.001
10 years mortality	0.003
**Clinical variables**	
Glycemia (mmol/L)	0.271
VO_2_max (mL/kg/min)	0.017
Hand grip (kg)	0.064
BMI (kg/m^2^)	0.682
FFM	0.449
**Comorbidities**	
Former cigarette smoking	0.889
Asthma	0.257
COPD	0.366
COPD exacerbation	0.883
IMA	0.632
Hypertension	0.599
Ischemic stroke	NV
Rheumatoid arthritis	0.583
Arthrosis	0.259
Neck pain	0.741
Cephalgia	0.093
Low back pain	0.396
DM	0.132
Allergy	0.117
Gastro duodenal ulcers	0.547
Liver cirrhosis	0.277
Cancer	0.810
Urinary disorders	0.093
Anxiety	0.065
Depression	0.101
Psychiatric disorders	0.176
Impairment post-major trauma	0.477
Metabolic disorders	0.982
**Therapy**	
Number of drugs taken	0.093
Diuretics	0.404
TP Dyslipidemia	0.492
TP Diabetes	0.265
TP Hypertension	0.074
TP Gout	0.904
NSAIDs	0.154
TP Ulcer	0.778
TP Anti-arrhythmias	0.060
TP Thyroid	0.048
TP Asthma/allergy	0.775
CCS	NV
Antidepressant	0.314
Other therapy	0.712

BMI = Body Mass Index; ABSI: a new body shape index; VO_2_Max = maximum oxygen consumption; FFM = free fat mass; COPD = Chronic obstructive pulmonary disease; IMA = myocardial infarction; CCS = systemic corticosteroids; DM = diabetes mellitus; NSAIDs = nonsteroidal anti-inflammatory drugs.

**Table 3:** ABSI correlation with VO_2_Max and systolic blood pressure. Nonparametric correlation index (^a^). Significance based on a two-tailed distribution (^b^). Lower and upper confidence intervals (^c^).

Correlation					
	Variable	Spearman Rho ^a^	*p*-Value ^b^	Lower C.I. ^c^	Upper C.I. ^c^
ABSI standardized	VO_2_Max	−0.190	0.017	−0.336	−0.035
	Systolic BP	0.262	<0.001	0.110	0.401

VO_2_Max = maximum oxygen consumption; Systolic BP = systolic blood pressure.

**Table 4:** Univariate survival analysis. Statistical significance (^a^).

	Non-Survivor N = 10	Survivor N = 149	*p*-Value ^a^
Female	4 (40)	90 (60)	0.319
Age (years)	67 (63–72)	67 (64–70)	0.878
**Clinical variables**			
Arterial systolic pressure (mmHg)	143 (134–153)	140 (130–158)	0.699
BMI (kg/m^2^)	28 (25–31)	25 (22–27)	0.033
Waist circumference (cm)	101 (87–112)	89 (82–94)	0.007
ABSI_std_	0.90 ± 0.84 (SD)	−0.060 ± 0.98 (SD)	0.005
Glycemia (mmol/L)	5.77 (5.26–6.06)	5.27 (4.87–5.61)	0.045
**Comorbidities**			
Former cigarette smoking	9 (90)	71 (48)	0.018
Asthma	0	8 (6.3)	1.000
Allergy	1 (11.1)	31 (24)	0.685
COPD	0	10 (7.8)	1.000
IMA	9	125	NV
Hypertension	3 (33.3)	44 (32.4)	1.000
Ischemic stroke	0	0	NV
Rheumatoid arthritis	0	14 (10.9)	1.000
Arthrosis	2 (20)	30 (23.3)	1.000
Neck pain	4 (40)	50 (38.5)	1.000
Cephalgia	1 (11.1)	33 (25.6)	0.465
Low back pain	0	12 (9.6)	1.000
DM	0	4 (3.1)	1.000
PUD	0	8 (7.3)	1.000
Liver cirrhosis	1	3 (2.4)	0.264
Cancer	4 (44.4)	12 (9.6)	0.012
Urinary disorders	0	27 (20.8)	0.206
Anxiety	0	12 (9.4)	1.000
Depression	0	3 (2.4)	1.000
Psychiatric disorders	1 (11.1)	0	0.067
Impairment post-major trauma	0	5 (4)	1.000
Metabolic disorders	1 (12.5)	29 (23.2)	0.683
**Therapy**			
Number of drugs taken	1 (0–1)	1 (0–2)	0.644
Diuretics	0	11 (7.4)	1.000
TP Dyslipidemia	0	23 (15.4)	0.360
TP Diabetes	0	2 (1.3)	1.000
TP Hypertension	3 (30)	42 (29)	1.000
TP Gout	0	1 (0.7)	1.000
NSAIDs	1 (10)	8 (5.4)	0.451
TP Ulcer	0	9 (6)	1.000
TP Anti-arrhythmias	3 (30)	7 (4.7)	0.017
TP Thyroid	0	11 (7.4)	1.000
TP Asthma/allergy	0	4 (2.7)	1.000
CCS	0	0	NV
Antidepressant	1 (10)	1 (0.7)	0.122
Other therapy	1 (10)	43 (28.9)	0.286

mmHg = millimeters mercury; ABSI_std_ = standardized ABSI; BMI = Body Mass Index; VO_2_Max = maximum oxygen consumption; FFM = free fat mass; COPD = chronic obstructive pulmonary disease; IMA = myocardial infarction; CCS = systemic corticosteroids; DM = diabetes mellitus; NSAIDs = nonsteroidal anti-inflammatory drugs.

**Table 5:** Logistic regression to determine 10-year mortality. Logistic regression coefficient (^a^). Standard error (^b^). Wald Test (^c^). Statistical significance (^d^). Adjusted odds ratio (^e^). Lower and upper confidence intervals (^f^).

	B ^a^	S.E. ^b^	Wald ^c^	*p*-Value ^d^	Odds Ratio ^e^	Lower C.I. ^f^	Upper C.I. ^f^
ABSI standardized	1.019	0.480	4.512	0.034	2.771	1.082	7.098
TP anti-arrhythmias	3.030	1.114	7.405	0.007	20.706	2.334	183.661
Cancer	3.009	0.998	9.902	0.003	20.272	2.867	143.339
Cigarette smoke (former)	3.147	1.286	5.985	0.014	23.260	1.870	289.386
K	−6.56	1.480	19.686	<0.001	0.001		

TP anti-arrhythmias = antiarrhythmic therapy; K = regression constant.

**Table 6:** Receiver Operating Characteristic statistics. Area under the curve (^a^). Standard error (^b^). Statistical significance (^c^). Lower/upper confidence intervals (^d^).

ROC Analysis					
	Area ^a^	S.E. ^b^	*p*-Value ^c^	Lower C.I. ^d^	Upper C.I. ^d^
ABSI standardized	0.766	0.081	0.001	0.607	0.925
ABSI logistic model	0.911	0.050	0.000	0.812	1.010

**Table 7:** Z statistic on the difference between the ROC curve planes. Z value (^a^). Statistical significance (^b^). Arithmetic difference between the curves (^c^). Standard error of AUC difference (^d^). Confidence intervals for AUC difference (^e^).

ROC Curve Difference						
	Z ^a^	*p*-Value ^b^	AUC Difference ^c^	S.E. diff. ^d^	Lower C.I. ^e^	Upper C.I. ^e^
ABSI standardized						
ABSI logistic model	−2.973	0.003	−0.145	0.353	−0.240	−0.049

**Table 8:** Hypothesis testing to compare the survival distributions of two samples. χ^2^ (^a^). Statistical significance (^b^).

	Chi-Square ^a^	*p*-Value ^b^
Log Rank (Mantel–Cox)	16.545	<0.001
Breslow (Gen. Wilcoxon)	16.532	<0.001
Tarone–Ware	16.539	<0.001

**Table 9:** Cox regression of 10-year mortality. Regression coefficient (^a^). Standard error (^b^). Likelihood ratio test (^c^). Statistical significance (^d^). Hazard ratio (^e^); lower and upper confidence interval of hazard ratio (^f^).

	B ^a^	S.E. ^b^	Wald ^c^	*p*-Value ^d^	Hazard Ratio ^e^	Lower ^f^	Upper ^f^
ABSI cutoff	1.537	0.739	4.324	0.038	4.650	1.092	19.793
TP anti-arrhythmias	2.629	0.864	9.255	0.002	13.862	2.548	75.411
Cancer	3.009	0.800	7.879	0.005	9.456	1.970	45.394
Cigarette smoke	3.147	1.121	4.927	0.026	12.047	1.338	108.474

**Table 10:** Male ABSI.

ABSI _std_ Male N°65	*p*-Value
**Demographic variables**	
LogGlycemia	0.333
LogBMI	0.575
Arterial systolic pressure	0.001
Log FFMI	0.834
VO_2_Max	0.001
Hand grip	0.028
**Comorbidity**	
Former cigarette smoking	0.628
Asthma	0.831
COPD	0.565
COPD exacerbation	0.636
IMA	0.343
Hypertension	0.259
Ischemic stroke	NV
Rheumatoid arthritis	0.739
Arthrosis	0.794
Neck pain	0.301
Cephalgia	0.271
Low back pain	0.983
DM	0.583
Allergy	0.985
Gastro duodenal ulcers	0.223
Liver cirrhosis	0.742
Cancer	0.193
Urinary disorders	0.989
Anxiety	NV
Depression	NV
Psychiatric disorders	0.188
Impairment post-major trauma	0.187
Metabolic disorders	0.323
**Therapy**	
Number of drugs taken	0.283
Diuretics	0.363
TP Dyslipidemia	0.265
TP Diabetes	0.383
TP Hypertension	0.603
TP Gout	0.833
NSAIDs	0.754
TP Ulcer	0.141
TP Anti-arrhythmia	0.101
CCS	NV
TP Thyroid	0.563
TP Asthma/allergy	NV
Antidepressant	0.178
Other therapy	0.915

LogGlycemia = natural logarithm of glycemia; LogBMI = natural logarithm of the Body Mass Index; VO_2_max = maximum oxygen consumption; LogFFMI = natural logarithm of free fat mass indexed by height; COPD = chronic obstructive pulmonary disease; IMA = myocardial infarction; CCS = systemic corticosteroids; DM = diabetes mellitus; NSAIDs = nonsteroidal anti-inflammatory drugs; NV = no value.

**Table 11:** Female ABSI.

ABSI_std_ Female N°94	*p*-Value
**Demographic variables**	
Age	0.005
Glycemia	0.173
LogBMI	0.382
Arterial systolic pressure	0.788
FFMI	0.905
VO_2_max	0.128
Hand grip	0.267
**Comorbidity**	
Former cigarette smoking	0.567
Asthma	0.519
COPD	0.414
COPD exacerbation	0.075
IMA	NV
Hypertension	0.345
Ischemic stroke	NV
Rheumatoid arthritis	0.373
Arthrosis	0.783
Neck pain	0.977
Cephalgia	0.325
Low back pain	0.231
DM	0.148
Allergy	0.308
Gastro duodenal ulcers	0.795
Liver cirrhosis	0.233
Cancer	0.342
Urinary disorders	0.181
Anxiety	0.293
Depression	0.236
Psychiatric disorders	NV
Impairment post-major trauma	0.897
Metabolic disorders	0.706
**Therapy**	
Number of drugs taken	0.201
Diuretics	0.588
TP Dyslipidemia	0.843
TP Diabetes	NV
TP Hypertension	0.148
TP Gout	NV
NSAIDs	0.278
TP Ulcer	0.567
TP Anti-arrhythmia	0.296
TP Thyroid	0.115
TP Asthma/allergy	0.517
CCS	NV
Antidepressant	0.818
Other Therapy	0.785

LogBMI = natural logarithm of the Body Mass Index; VO_2_max = maximum oxygen consumption; FFMI = free fat mass indexed by height; COPD = chronic obstructive pulmonary disease; IMA = myocardial infarction; CCS = systemic corticosteroids; DM = diabetes mellitus; NSAIDs = nonsteroidal anti-inflammatory drugs; NV = no value.

## Data Availability

The raw data supporting the conclusions of this article will be made available by the authors on request.
